# Active video games as a tool to prevent excessive weight gain in adolescents: rationale, design and methods of a randomized controlled trial

**DOI:** 10.1186/1471-2458-14-275

**Published:** 2014-03-24

**Authors:** Monique Simons, Mai JM Chinapaw, Maaike van de Bovenkamp, Michiel R de Boer, Jacob C Seidell, Johannes Brug, Emely de Vet

**Affiliations:** 1 Department of Health Sciences and the EMGO Institute for Health and Care Research, Faculty of Earth and Life Sciences, VU University Amsterdam, De Boelelaan 1085, 1081 HV Amsterdam, The Netherlands; 2 Body@Work, Research Center Physical Activity, Work and Health, TNO- VU/VUmc, VU University Medical Center, Amsterdam, The Netherlands; 3 TNO, Expertise Centre Life Style, Leiden, The Netherlands; 4 Department of Public and Occupational Health and the EMGO Institute for Health and Care Research, VU University Medical Center, Amsterdam, The Netherlands; 5 Department of Epidemiology & Biostatistics and EMGO Institute for Health and Care Research, VU University Medical Center, Amsterdam, The Netherlands; 6 Chairgroup Strategic Communication, Sub-department Communication, Philosophy and Technology: Centre for Integrative Development, Wageningen University and Research Centre, Wageningen, The Netherlands

**Keywords:** Video games, Active games, Exergames, Physical activity, Sedentary lifestyle, Adolescent, Prevention, Excessive body weight, Overweight, Randomized controlled trial

## Abstract

**Background:**

Excessive body weight, low physical activity and excessive sedentary time in youth are major public health concerns. A new generation of video games, the ones that require physical activity to play the games –i.e. active games- may be a promising alternative to traditional non-active games to promote physical activity and reduce sedentary behaviors in youth. The aim of this manuscript is to describe the design of a study evaluating the effects of a family oriented active game intervention, incorporating several motivational elements, on anthropometrics and health behaviors in adolescents.

**Methods/Design:**

The study is a randomized controlled trial (RCT), with non-active gaming adolescents aged 12 – 16 years old randomly allocated to a ten month intervention (receiving active games, as well as an encouragement to play) or a waiting-list control group (receiving active games after the intervention period). Primary outcomes are adolescents’ measured BMI-SDS (SDS = adjusted for mean standard deviation score), waist circumference-SDS, hip circumference and sum of skinfolds. Secondary outcomes are adolescents’ self-reported time spent playing active and non-active games, other sedentary activities and consumption of sugar-sweetened beverages. In addition, a process evaluation is conducted, assessing the sustainability of the active games, enjoyment, perceived competence, perceived barriers for active game play, game context, injuries from active game play, activity replacement and intention to continue playing the active games.

**Discussion:**

This is the first adequately powered RCT including normal weight adolescents, evaluating a reasonably long period of provision of and exposure to active games. Next, strong elements are the incorporating motivational elements for active game play and a comprehensive process evaluation. This trial will provide evidence regarding the potential contribution of active games in prevention of excessive weight gain in adolescents.

**Trial registration:**

Dutch Trial register NTR3228.

## Background

Excessive body weight, low physical activity and excessive sedentary time in youth are major public health concerns [1-3], especially among children and adolescents from lower educated [4] and ethnic minority parents [5]. Obesity is associated with an increased risk for a range of chronic diseases and presents a major burden of disease and health care spending [6-8]. One of the life stages that may play a critical role in the development and persistence of obesity and related co-morbidities into adulthood is adolescence [9,10]. The adolescent period is characterized by low physical activity, high levels of sedentary behavior and changes in body composition (amount and location of body fat), making adolescents an important target group for preventive strategies [9].

Overweight and obesity generally result from a long-term imbalance between energy intake (determined by dietary intake) and energy expenditure (mainly determined by physical activity and sedentary behaviors) [11]. Regarding the latter, sedentary activities such as watching TV and playing video games, have been found to be associated with negative health outcomes such as overweight and obesity, partly independent of diet and moderate to vigorous physical activity [12-16]. It has been suggested that reductions in sedentary behavior may be as effective as or even more effective than increasing physical activity directly in decreasing BMI, and percentage overweight [17]. A major and increasing contributor to sedentary behavior is playing video games, a wide spread and popular activity among adolescents [18-20]. In the USA, an average of almost two hours per day playing video games was reported for 8–18 year olds, with an increase of almost one hour per day from 1999 to 2009 [18]. Recent evidence indicates that in the Netherlands, about 80% of the adolescents (12–16 years old) play video games regularly and play on average five days a week with an average duration of 65 minutes on a school day and 80 minutes on a weekend day [19]. In the Netherlands time spent on playing video games seems to peak during adolescence [20,21].

Reducing time spent in sedentary video games (hereafter called non-active games) may thus contribute to reducing sedentary time and prevention of overweight and obesity in adolescents. A new generation of video games, the ones that require physical activity to play the games –i.e. active games– seems a promising alternative to traditional non-active games to promote PA and reduce sedentary behaviors in youth. These active games, such as provided by and on Nintendo Wii, Microsoft Kinect, and Sony PlayStation Move, require whole-body movement to play the video game instead of the small movements of fingers and wrists used in non- active gaming [22]. Results from various studies have suggested that energy expenditure may be substantially increased when playing active games compared to energy expenditure during non-active gaming and other sedentary screen activities such as watching television [23,24]. Active games can generally elicit light to moderate intensity physical activity (2–6 metabolic equivalents; METS) [23,24]. Consequently, when adolescents substitute the time they spent playing non-active games by playing active games, sedentary time will be reduced and physical activity increased, which may help to prevent excessive weight gain.

The potential strength of these active games is based on the intrinsic motivation of children to play video games. Intrinsic motivation is a construct based on the Self Determination Theory (SDT) and means that one enacts in an activity because one enjoys the activity and not because of external factors [25]. SDT posits that if one is intrinsically motivated, behavior change is more likely to be maintained [26]. Previous studies revealed that playing active games are perceived as fun and enjoyable [22]. Further, adolescents attending a low level of education are more likely to play active games frequently than those attending a higher level [19]. These results suggest that active games seem promising for reaching adolescents at risk, because they spend more time on non-active gaming and other sedentary behaviors [4,20] and are more likely to be overweight or obese [4].

Irrespective of energy expenditure, it can be hypothesized that active games may influence energy balance through reduced energy intake. Energy intake, especially the intake of snacks and sugar-sweetened beverages, is positively associated with time spent in sedentary screen activities such as watching TV en playing non-active games [16,27-30]. If active games can result in a decrease in sedentary time, it might simultaneously result in a lower energy intake. It is hypothesised that higher energy intake during sedentary activities is because they distract children from satiety cues [31]. It is possible that playing active games is less distractive than watching TV en playing non-active games [32]. On the other hand, it might also be that the physical activity required for playing active games influences appetite control [33] or may be thirst, resulting in a higher energy intake. However, up until now, little is known about energy intake during active gaming. So far only two studies have evaluated energy intake during playing active games [32,34]. Both studies showed no difference in energy intake while playing active games compared to energy intake while playing non-active games. However both studies were controlled laboratory studies and the effects of playing active at home on energy intake need to be further investigated. One randomized controlled trial with a 24-week follow-up showed a non-significant trend towards a decrease in self-reported energy intake from snacks in a home-based active gaming intervention group compared to a non-active game control group [35].

Substituting the time spent playing non-active games with playing active video games may thus reduce sedentary time, increase physical activity, reduce snacking and thereby prevent excessive weight gain in adolescents. So far only few and small scale studies have been conducted indicating that active games can decrease sedentary behavior and increase physical activity in youngsters on the short term (8 to 12 weeks) [36-39]. Other studies show that ongoing participation in active games is a problem [40,41]. To date only one well powered randomized controlled trial (RCT) on the effects of an active gaming intervention on body composition with a longer follow-up period has been conducted [35]. This trial showed a small effect of a six-months active game intervention on Body Mass Index (BMI) and body composition in 10–14 year old overweight/obese children. More evidence is needed from adequately powered studies, also including normal weight children, monitoring long term participation in the active games, and investigating long term effects [23,42].

Therefore, the current study aims to evaluate an active game intervention that incorporates motivational elements for adherence, includes a long intervention period and a comprehensive process evaluation. The intervention of the current study has four unique features that make this study worthwhile. First, so far no active game interventions were family-oriented. Parental involvement in interventions for children and adolescents has been recommended, therefore the current trial includes the family instead of only the individual adolescent [43]. Furthermore, games are often played at home and it has been recommended to change the obesogenic home environment to better facilitate and enable weight gain preventive behaviors [43]. Secondly, the current study includes several elements to promote active game play based on previous studies, such as encouraging social play and providing a variety of active games [22,44]. This is important because previous studies showed that maintaining participation in active game participation is difficult to achieve when providing active games without any additional efforts to encourage use [37,40,41]. Thirdly, previous studies mainly focused on the first generation of active games such as Dance Dance Revolution and PlayStation2®EyeToy [35-37,40,41], and as the gaming industry is evolving fast, there is a need for studies evaluating the newest generation of active games such as PlayStation® Move or Kinect games. Fourthly, to our knowledge the current study includes the longest follow up period (ten months) for an active game intervention.

The current manuscript describes the design of a trial evaluating the effects of a family-oriented active game intervention, incorporating several motivational elements. The overall study focuses on both the adolescents as well as his or her family members. The current manuscript focuses on the measurements and effectiveness among adolescents. The intervention consists of a) providing an upgrade package for the newest generation of active games (PlayStation®Move), b) an encouragement to replace at least 1 hour of non-active gaming by active gaming on the PlayStation Move, c) stimulating social play by providing an extra Move controller, and d) stimulating long term use by providing multiple active games.

We will test the following hypotheses:

1) The intervention will result in lower BMI-SDS (SDS = adjusted for mean standard deviation score using the 1997 Dutch growth study as reference), waist circumference-SDS, hip circumference and skinfold thickness compared to the control condition after four and ten months.

2) The intervention will lead to less self-reported time on sedentary screen activities compared to the control condition after four and ten months.

3) The intervention will lead to less self-reported sugar-sweetened beverages and snacks consumption compared to the control condition after four and ten months.

## Methods/Design

### Design

The efficacy of the active game intervention is evaluated in a randomized controlled trial among adolescents aged 12 to 16 years old and their families. Families are randomly assigned to the intervention or control group after baseline assessment by the researcher or research assistant using a pre-determined randomization list. The randomization list consists of a row with the numbers one and two in a random order and is based on a block randomization scheme, with blocks of 100, obtained from an online computer program (http://www.randomization.com). Participants are assigned to one of the research groups based on this randomization list in order of checking out after they finish the baseline measures. The intervention group is exposed to the active game intervention and the control group is asked to continue their normal game play behavior and they receive the material belonging to the intervention at the end of the study. Measures are taken at baseline (T_0_) (adolescent anthropometrics and questionnaire), after one month (T_1m_) adolescent questionnaire (including process evaluation for intervention group adolescents), four months (T_4m_) adolescent anthropometrics and questionnaire (including process evaluation for intervention group adolescents); and ten months (T_10m_) adolescent anthropometrics and questionnaire (including process evaluation for intervention group adolescents) (Table 1). Primary outcomes are adolescents’ measured BMI-SDS (SDS = adjusted for mean standard deviation score), waist circumference-SDS, hip circumference and skinfolds thickness. Secondary outcomes are adolescents’ self-reported sedentary behavior, and consumption of sugar-sweetened beverages and snacks. Additionally, physical activity behavior excluding active game play is assessed to check whether the active game intervention was at the expense of physical activity.

**Table 1:** Design scheme

	**Measure moment**			
**Measures**	**T**_**0**_	***T***_***1m***_	***T***_***4m***_	***T***_***10m***_
***Anthropometrics***				
(Weight, height, waist and hip circumference, skinfolds)	x		x	x
***Questionnaires***				
Time spent active and non-active gaming	x	x	x	x
Physical activity and sedentary screen time	x	x	x	x
Consumption of snacks and sugar sweetened beverages	x	x	x	x
Companions for active/non-active gaming				x
Game consoles and applications owned	x	x	x	x
***Process evaluation (only in intervention group)***				
**1) Adherence to the intervention**				
Usage of Move games		x	x	x
Reasons for not playing (the minimal of one hour per week)		x	x	x
**2) Opinion of the Move games and the intervention**				
Enjoyment with respect to playing the Move games		x	x	x
Most played Move game		x	x	x
Most enjoying Move game		x	x	x
Ease of use		x	x	x
Perceived competence		x	x	x
Perceived physical exertion of playing Move games				x
Opinion on the amount of provided Move games				x
Self purchased Move game				x
**3) Game context**				
Move game companions				x
Location of PlayStation Move console				x
**4) Potential adverse effects** (occurrence of injuries because of playing with the Move games)				x
**5) Activity replacement**			x	x
**6) Intention to continue playing the Move games**				x

*T*_*0*_, Baseline measurement; *T*_*1m*_, Measurement one month after baseline; *T*_*4m*_, Measurement four months after baseline; *T*_*10m*_, Measurement ten months after baseline.

The study was approved by the Medical Ethics Committee of the VU Medical Centre.

### Blinding

It is not possible to perform the randomization blind, because based on the randomization the adolescents receive an active game upgrade package or not. At T_0_ the measures are conducted blind for participants and research assistants regarding group assignment. At the end of the T_0_ measure the randomization takes place and therefore the measures at T_4m_ and T_10m_ cannot be conducted blind for participants or assessors. The intervention group receives new games at T_4m_ and the control group receives their Move package at T_10m_. Data analyses are not conducted blind.

### Participants

The current study focuses on adolescents aged 12–16, who are the main participants of the study. Additionally the family members of the adolescent (parents and siblings aged 8–18 years old) are included in the study but they are not used for current analyses. Inclusion criteria are:

•Adolescent plays ≥ 2 hours of non-active video games per week

•Adolescent does not play active games yet, or less than once a week.

•Adolescent is physically and mentally able to play active games

•Adolescent has access to a PlayStation3 in his or her home.

•Family does not have a Move upgrade for PlayStation3

•Adolescent lives in the participating household (assuring sufficient access to the Move games provided as part of the intervention) at least 4 days a week.

•At least one family member (parent or sibling aged 8–18 years old) is willing to participate in the study (filling in questionnaires).

### Sample size

Sample size calculation is performed to estimate how many adolescents should be included to detect a significant difference of 0.5 kg excess body weight between the intervention and control condition during the follow-up measurements (primary analysis). The 0.5 kg excessive weight gain is based on calculations in adults, because calculations for adolescents were lacking at that time. Adult individuals may gain on average 0.5 kg excess body weight per year, which results from an energy imbalance of only 70 Kcal per week per year [43]. We computed, based on energy expenditure studies, that an excess weight gain of 0.5 kg, may be prevented by substituting one hour per week of non-active gaming by playing active games [45]. To have sufficient power to detect a clinically relevant difference in excess weight gain of 0.5 kg (SD = 1.5 kg) between the intervention and control condition during follow up, with a power of 0.80, alpha .05 and an intraclass correlation coefficient (ICC) for within-subject clustering of observations of 0.7, 99 participants in each condition are needed. Taking into account an anticipated drop-out of 20%, a total of 119 adolescents per condition needs to be recruited.

### Recruitment

Recruitment of adolescents takes place in four cities in the Netherlands: Amsterdam, Amersfoort, Leiden and Breda. Advertisements are posted on game websites, game magazines, Facebook, newsletter for PlayStation owners and local papers, and announcements are made on local radio. In addition, local health organizations (GGD) and municipalities in the four cities collaborate by providing addresses of families with a child aged 12–16 years old. In Leiden all families with a 12–16 years old child receive an invitation letter, and in Amsterdam, Breda and Amersfoort we make a selection based on postal code areas covering neighborhoods with a relatively low social economic status. Also, flyers are distributed in schools and popular places for gaming adolescents (e.g. game shops, music stores). Interested adolescents and families can provide their contact details on our project website or send it by mail. The interested families are contacted by email and asked to complete an online questionnaire to assess further inclusion criteria. Next, eligible families receive information about participation and an informed consent form that they have to fill in prior to the baseline measure. Then, families receive information about the baseline online questionnaires and an invitation for the adolescent’s baseline assessment.

### Intervention

Adolescents assigned to the intervention group receive a PlayStation®Move upgrade package to play active games on a PlayStation3 console. PlayStation®Move uses a handheld motion controller wand and a motion-capture PlayStation®Eye camera that tracks the position, and inertial sensors in the wand to detect its motion. In this way every movement of the player is mimicked on-screen in the game. Although, there are multiple active game systems (such as Nintendo Wii, Dance Dance Revolution, Microsoft Xbox Kinect, PlayStation2®EyeToy) available on the market, the PlayStation®Move was chosen for the current study for several reasons. First, a survey study showed that the PlayStation was the most frequently owned game console among 12–16 year old adolescents [19]. So by choosing an application that is compatible with the PlayStation, we optimize the chances for recruiting sufficient participants. Second, the active game system for the PlayStation is one of the newest on the market at time of the study, making use of the most sophisticated technology, and may be more affordable (around €50) than other consoles or active game upgrades (e.g. Microsoft Kinect (around €135)).

As mentioned in the introduction previous studies showed that attention should be paid to (ongoing) participation in active game play, therefore we include several motivation enhancing intervention elements. Since variation in games is important [22,46,47], families receive four active Move games (Sport Champions, Move Fitness, Start the Party and Medieval Moves) at the start of the study and two more (Dance Star Party and Sorcery) after four months. Social and family play is important [22,44], and we provide two controllers to promote playing together with family and friends. Further, research has shown that providing instruction to use the active games seems an important addition to providing the games for physical activity promotion [46,48], therefore we explicitly encourage participants at each contact moment to substitute non-active gaming with active gaming as much as possible but at least one hour per week. One hour per week corresponds with approximately 70 kcal (representing the energy imbalance that can result in excessive weight gain) [43], and was regarded as a feasible change.

Adolescents in the control group are asked to continue their normal gaming behavior. They receive a PlayStation Move starters pack at the end of the study as an incentive for their participation. Further, they receive a small gift (e.g. magazine, lanyard, pen) as an incentive after participation at each measure moment.

### Measurements

#### Adolescents’ anthropometrics (T_0_, T_4m_, T_10m_) (primary outcomes)

Adolescent’s anthropometric measures: height, weight, waist and hip circumferences and skinfolds thickness are performed at T_0_, T_4m_ and T_10m_ on pre-scheduled days at central places in the same four cities where we recruited the participants. These measurements take place at a site close to where the participants live (a museum, school or soccer stadium). Adolescents who are incapable to visit during the pre-scheduled days are measured at home. Trained research assistants conduct the measurements in an accurate manner according to a standardized protocol.

Height is measured to the nearest 0.1 cm using a Seca type 214 stadiometer with adolescents standing on bare feet and their head in the Frankfort plane. Weight is measured on bare feet with a calibrated electronic scale (SECA) with an accuracy of 0.1 kg. Adolescents are allowed to wear light indoor clothing, such as a t-shirt and shorts/short pants, and are asked to remove belt or any other heavy objects of use such as phone, keys or wallet. BMI (kg/m^2^) is calculated by dividing weight (kg) by height squared (m^2^). Next, BMI-SDS is determined using the data from the fourth Dutch growth study among children of 1997 as reference [3] employing the Growth Analyser software (https://www.growthanalyser.org/). Waist and hip circumference are measured with a tape measure (brand Jobst) to the nearest 0.1 cm. For waist circumference we also determine the SDS values using the same reference group and software as for BMI. Skinfolds are measured with an accuracy of 0.2 mm using four-site skinfold measures (triceps, biceps, subscapular and suprailiac) with a Harpenden skinfold calliper [49]. For hip circumference and skinfold thickness valid Dutch reference data to calculate SDS scores are not available.

At least two readings of each measurement (weight, height, waist/hip circumference and skinfolds) are obtained to deal with intra-observer variability, and the mean of the two measurements is used for analyses. If the two readings differ more than 1 cm for height, more than 0.2 kg for weight, more than 1 cm for waist and hip circumference, more than 1 mm for skinfolds, a third measurement is taken. In case of three measurements, the outlier of the three will be excluded.

#### Self-reported measures

Online questionnaires (made with the online tool NETQ http://www.netq-enquete.nl/nl) are emailed to the adolescents at T_0_, T_1m_, T_4m_ and T_10m_. The questionnaires assess demographics birth date, sex, educational level (pre-vocational; higher continued education; pre-university) and country of birth. Also active and non-active gaming behavior, other energy balance related behaviors, game consoles/application owned and game companions are assessed in the questionnaires (Table 2). Among the adolescents in the intervention group a process evaluation is conducted. Several process evaluation questions are added in the questionnaire of the intervention group at T_1m_, T_4m_, T_10m_ (Table 3). Furthermore, adolescents in the intervention group are asked to report their use of the Move games over the entire ten-month period using a calendar format.

**Table 2:** Gaming aspects and behavior, physical activity, sedentary and dietary behavior assessed in questionnaires

	**Items and answering categories**	**Instrument/source**
**Gaming aspects and behavior**		
Time spent non-active gaming	*1. How often do you play non-active games on weekdays? (1–5 days a week)*	De Jong et al., 2013 [50]
	*2. How long per day do you play non-active games on a weekday? (… hours; … minutes)*	
	*3. How often do you play non-active games on weekend days? (1–2 days a week)*	
	*4. How long per day do you play non-active games on a weekend day? (… hours; … minutes)*	
Time spent active gaming	*1. How often do you play active games on weekdays? (1–5 days a week)*	De Jong et al., 2013 [50]
	*2. How often do you play active games on weekend days? (1–2 days a week)*	
	*3. How often do you play active games on weekend days? (1–2 days a week)*	
	*4. How long per day do you play active games on a weekend day? (… hours; … minutes)*	
Companions for active/non-active gaming	*With whom do you usually play active/ non-active games?*	Specifically developed for the current study^1^
	- *By myself,*	
	- *With my parents,*	
	- *With my brother/sister,*	
	- *With my friends,*	
	- *With somebody else, namely …*	
Game consoles and applications owned	*Which consoles and application do you have at home? (PlayStation 1/2/3; PlayStation Move; PSP (PlayStation Portable); Xbox; Xbox 360; Kinect; (Super)Nintendo; Nintendo Wii; Nintendo DS(i); Gamecube; Gameboy; Dance Dance Revolution; EyeToy; Computer/Laptop; iPad or another tablet)*	Specifically developed for the current study^1^
**Physical activity**		
Sports	*1. Which sports do you play in leisure time?*	Flemish physical activity computer questionnaires [51]
	*2. How often do you play these sports in leisure time? (1–7 times a week, > 7 times a week)*	
	*3. How long do you play these sports when you play them in leisure time (…hours; ..minutes)*	
**Active transport**			
*School*	*1. How do you usually go to school? (bike, walking, train/car/bus/scooter/moped, step/roller-skates/skateboard/wave board)*	Flemish physical activity computer questionnaires [51]	
	*2. How long does it take to go from home to school (one way)? … minutes*		
	*3. Do you go home during lunch break? (yes; no)*		
	*4. On which days do you go home during*		
	*5. Lunch break? (Monday; Tuesday; Wednesday; Thursday; Friday)*		
*Leisure time*	*Weekdays*		
	*6. How long on a weekday do you usually walk in your leisure time to go somewhere (excluding walking to school, work or trainee post)? (0–10; 10–20; 20–30; 30–40;40–50; 50–60 minutes per day; more than one hour per day (… hours and … minutes per day)*		
	*7. How long on a weekday do you usually cycle in your leisure time to go somewhere (excluding walking to school, work or trainee post)? (0–10;10–20; 20–30; 30–40; 40–50; 50–60 minutes per day; more than one hour per day (… hours and … minutes per day)*		
	*Weekend days*		
	*8. How long on a weekend day do you usually walk in your leisure time to go somewhere (excluding walking to school, work or trainee post)? (0–10; 10–20; 20–30; 30–40; 40–50; 50–60 minutes per day; more than one hour per day (… hours and … minutes per day)*		
	*9. How long on a weekend day do you usually cycle in your leisure time to go somewhere (excluding walking to school, work or trainee post)? (0–10; 10–20; 20–30; 30–40; 40–50; 50–60 minutes per day; more than one hour per day (… hours and … minutes per day)*		
**Sedentary screen time**			
TV time	*About how many hours a day do you watch television in a normal week? (excluding school time)*	Flemish physical activity computer questionnaires [51]	
	- *During weekdays: …. hours; …. minutes.*		
	- *During weekend days: …hours; …. minutes*		
Computer time	*About how many hours a day do you spend on the computer (e.g. chatting, internet) in a normal week? (excluding non-active and active gaming)*		
	- *During weekdays: …. hours; …. minutes.*		
	- *During weekend days: …hours; …. minutes*		
**Dietary behavior**			
Consumption of sugar sweetened beverages	Sugar-sweetened beverages were defined as carbonated soft drinks, other non-carbonated sugar sweetened drinks (water-based beverages that contain sugar) and so-called sport and energy drinks (e.g. AA, Extran, Aquarius, Red Bull). Excluding light or diet drinks and fruit juices.	Van der Horst et al. 2008 [52]	
	1. *On how many days a week do you usually drink soda? (1–7 days a week)*		
	2. *If you drink soda, how many soda do you drink on average on one day?*		
	*How many glasses/packages (250 ml)? (none, 1, 2, 3, 4, 5, 6, 7 or more)*		
	*How many cans (330 ml)? (none, 1, 2, 3, 4, 5, 6, 7 or more)*		
	*How many bottles (500 ml)? (none, 1, 2, 3, 4, 5, 6, 7 or more)*		
	*3. On how many days a week do you usually eat cake or candy? (1–7 days a week)*		
	*4. If you eat cake or candy, how many do you eat on average on one day?*		
	*1, 2, 3, 4, 5, 6 or more portions/pieces*		
Consumption of snacks	Savory (fast-food, pizza, fries, chips, nuts).	Van der Horst et al. 2008; Van Assema et al., 2001 [52,53]	
	1. *On how many days a week do you usually eat snacks?*		
	*1-7 days a week*		
	*5. If you eat snacks, how many do you eat on average on one day?*		
	*1, 2, 3, 4, 5, 6 or more portions/pieces*		
	Sweet (candy, candy bars, chocolate, cake, biscuits)		
	1. *On how many days a week do you usually eat cake or candy?*		
	*1-7 days a week*		
	*2. If you eat cake or candy, how many do you eat on average on one day?*		
	*1, 2, 3, 4, 5, 6 or more portions/pieces*		

^1^The questions that were specifically developed for the current study were based on focus groups and/or a previous survey study on active and non-active gaming among adolescents [19,22] and were pilot-tested in gaming adolescents to assure understanding and face validity.

**Table 3:** Process evaluation measures assessed in intervention group

**Process evaluation concepts**	**Items and answering categories**	**Instrument/source**
**1) Adherence to the intervention**		
Usage of Move games	*Did you succeed in playing the move games for at least one hour per week?*	Specifically developed for the current study^1^
	-* Yes, I played the move games for at least one hour per week*	
	- *No, In some weeks I failed to play the move games for at least one hour*	
	- *No, I never succeed in playing the move games for at least one hour per week*	
Reasons for not playing (the minimal of one hour per week)	*Why did you not managed to play the move games for at least one hour per week?*	Specifically developed for the current study^1^
	- *I was too hot*	
	- *I was not allowed by my parents*	
	- *There was somebody else playing on the Move.*	
	- *The move console did not worked properly*	
	- *The move games did not respond well to my movements*	
	- *I think the move games are too tiring*	
	- *I think the move games are boring*	
	- *We have not enough space to play to move games well*	
	- *I had not enough move games*	
	- *I did not have time*	
	- *I think the move games are stupid*	
	- *I had to many other things to do*	
	- *There wasn’t somebody to play with and it is boring to play the games by myself*	
	- *I was injured*	
	- *I rather play non active games*	
	- *Other, namely …*	
**2) Opinion of the move games and the intervention**		
Enjoyment with respect to playing the Move games	*I enjoy playing the Move games (totally disagree (1) – totally agree (7))*	Intrinsic Motivation Inventory (sub scale enjoyment) [54]
	*Playing the Move games is fun to do (totally disagree (1) – totally agree (7))*	
	*I think playing the Move game is boring (totally disagree (1) – totally agree (7))*	
	*Playing the Move games could hold my attention (totally disagree (1) – totally agree (7))*	
	*I would describe playing Move games as very interesting (totally disagree (1) – totally agree (7))*	
	*I think playing the Move games is quite enjoyable (totally disagree (1) – totally agree (7))*	
	*While playing the Move games, I was thinking about how much I enjoyed it (totally disagree (1) – totally agree(7))*	
Most played move game	*Which game did you play most often?*	Specifically developed for the current study^1^
Most enjoying move game	*Which game did you like the most?*	Specifically developed for the current study^1^
Ease of use	*It’s easy for me to learn how the Move games work (totally disagree (1) – totally agree (5))*	Davis, 1989; Hsu & Lu, 2004 [55,56]
	*Playing the Move games is easy for me (totally disagree (1) – totally agree (5))*	
Perceived competence	*I believe I am good in playing Move games (totally disagree (1) – totally agree (5))*	Intrinsic motivation inventory (subscale competence) [54]
	*I think I am better at playing the Move games than other people of my age and gender (totally disagree (1) – totally agree (5))*	
	*I am generally happy with my gaming performance (totally disagree (1) – totally agree (5))*	
Perceived physical exertion of playing move games	*How would you rate the intensity level of playing the Move games?*	Specifically developed for the current study^1^
	- *Light, little body movements are required while playing on the Move, and my breath rate does not accelerate;*	
	- *Moderate, I move my body quite a lot while playing on the Move and my breath rate accelerates a little bit;*	
	- *Hard, I have to move a lot and fast, I get out of breath and I am sweating during playing on the Move*	
Opinion on the amount of provided Move games	*What do you think about the amount of provided move games? (too little - exactly right - too much)*	Specifically developed for the current study^1^
Self purchased move game	*Did you bought, received, borrowed or downloaded other Move games in addition to the games we provided you?*	Specifically developed for the current study^1^
**3) Game context**		
Move game companion	*With whom do you usually play the Move games? (by myself, with my parents, with my brother/sister, with my friends, with somebody else, namely …)*	Specifically developed for the current study^1^
Location of PlayStation move console	*Where was the PlayStation PlayStation®Move located most of the time? (Living room, my own bedroom, my brother’s or sister’s bedroom, in the attic, in a shared game room, somewhere else, namely …)*	Specifically developed for the current study^1^
**4) Potential adverse effects**		
Occurrence of injuries because of playing with the move games	*Did you hurt or injured yourself during playing with the Move? (yes; no)*	Specifically developed for the current study^1^
	*What kind of injury occurred during game play? (bruise; graze; strained muscles or tendons; bruised something (e.g. ankle, wrist); broke something (e.g. leg, wrist, arm); some other injury, namely ….)*	
**5) Activity replacement**	*Can you think back about the time you did not have the Move games yet, what did you do with the time you now spend on active gaming?*	Simons et al., (2012) [45]
**6) Intention to continue playing the move games**	*1. I intend to continue playing the Move games;*	Based on Theory of Planned Behavior [57]
	*2. I expect to continue with playing the Move games (totally disagree (1) – totally agree (5)).*	

^1^The questions that were specifically developed for the current study were based on focus groups and/or a previous survey study on active and non-active gaming among adolescents [19,22] and were pilot-tested in gaming adolescents to assure understanding and face validity.

##### Time spent active and non-active gaming (T_0_, T_1m_, T_4m_, T_10m_)

To assess time spent active and non-active gaming, questions are formulated based on existing and validated questionnaires for adolescents asking about frequency and duration, for school and weekend days separately [50,58]. Adolescents can indicate the duration by selecting one of four categories (<30 minutes, 30–60 minutes, 1–2 hours, and >2 hours). Average values are calculated for every category (<30 minutes = 15 minutes; 30–60 minutes = 45 minutes; 1–2 hours = 90 minutes; >2 hours = 150 minutes) and multiplied by the frequency, resulting in total hours per week spent on active and non-active games separately.

##### Other physical activity and sedentary screen time (T_0_, T_1m_, T_4m_, T_10m_)

Assessment of physical activity and sedentary screen time occurs by determining the frequency and the amount of time spent on different leisure-time sedentary and physical activities based on the validated Flemish Physical Activity Computer Questionnaires (FPACQ) [51]. The FPACQ assesses physical activity and sedentary behavior in multiple domains: school activity (physical activity and sports participation at school excluding physical education lessons), active transport (transportation for example by bike, and walking (for both school and leisure time), non-active transport (transportation for example by car or bus (for both school and leisure time) leisure time sports participation, and sedentary activities (watching television and computer activities). For the current study we focus on the domains active transport and leisure time sports participation for physical activity. Physical activity outcomes are leisure time sport behavior (MET*hours per week), active transport to school (minutes per week) and walking and cycling in leisure time (minutes per week). Sports participation in MET*hours per week is calculated by multiplying the reported hours per week by their corresponding MET values according to the compendium of MET values [59]. For sedentary screen time we focus on computer time (hours per week) and TV time (hours per week). Computer time includes all activities on a computer such as chatting and surfing on the internet, except playing computer games. Both computer and TV time are assessed separately for week- and weekend days.

##### Consumption of snacks and sugar sweetened beverages (T_0_, T_1m_, T_4m_, T_10m_)

Consumption of sugar-sweetened beverages is assessed using questionnaires by Van der Horst et al. [52]. Consumption of sugar-sweetened beverages includes consumed glasses, cans and bottles of carbonated and non-carbonated soft drinks, lemonade, and sport and energy drinks. Diet sodas and juices are not assessed. Total consumption of sugar sweetened beverages is expressed in liters per day, and is calculated according to Dutch standard serving sizes (1 glass = 200 ml, 1 can = 330 ml, 1 bottle = 500 ml). Consumption of snacks is assessed using items from a validated questionnaire [52,53]. Snacks are classified as savory (e.g. fast-food, pizza, fries, chips, nuts) or sweet (e.g. candy, candy bars, chocolate, cake, biscuits) eaten between the main meals, and not as side servings at main meals. Quantity of consumed snacks is obtained by determining amount of “snack days” and amount of snacks consumed per “snack day”. These questions are combined into a single score for the amount of portions snack intake per week.

##### Game consoles and applications owned (T_0_, T_1m_, T_4m_, T_10m_)

At all measurements we assess which game consoles and game application the adolescents have access to in their home.

##### Game companions for active/non-active gaming (T_10m_)

At T_10m_ we assess with whom the adolescents usually play active and non-active games.

##### Process evaluation measures

A comprehensive process evaluation is conducted in the intervention group at T_1m_, T_4m_, T_10m_. Findings from focus groups and a survey about active and non-active gaming in adolescents provided a rationale for evaluating the following six elements [19,22]: 1) adherence to the intervention (usage of Move games, reasons for not playing at least one hour per week), 2) opinion of the Move games and the intervention (enjoyment, most played Move game, most enjoying Move game, ease of use, perceived competence, perceived physical exertion of playing Move games, opinion on the amount of provided Move games, self purchased Move game), 3) Game context (Move game companions, location of PlayStation Move console), 4) potential adverse effects (occurrence of injuries because of playing with the Move games), 5) activity replacement and 6) intention to continue playing the Move games. Table 3 shows all the process evaluation concepts, the items and answering categories as assessed in the questionnaire.

In addition to the questionnaires, adolescents’ time spent playing the active Move games in the intervention group is assessed on a daily basis over the whole 10-month period using a calendar format. The adolescents are asked to report type of Move game and amount of hours and minutes spent playing the Move games, according to Chinapaw et al. [41].

### Statistical analyses

First descriptive analyses are performed and data are checked for a normal distribution. Subsequently, control group and intervention group are described on baseline for demographics and outcome measures. Next, the effects of the intervention on all outcomes are evaluated by multilevel analyses. In the primary analyses, we analyze those participants which have at least one follow-up measurement in the groups they were randomized to. As a sensitivity analyses we will also impute follow-up measurements for persons with only a baseline measurement using multiple imputation with chained equations (intention to treat). Two models are constructed for each outcome measure: *model 1* adjusts for the baseline value of the outcome measure; and *model 2* additionally adjusts for demographics (age, sex, ethnicity and adolescent educational level) that are expected to associate with the outcome [3-5]. Second, analyses are performed according to the per protocol principle including the participants in the intervention group who played the Move games at least one hour per week. Adherence to protocol will be defined for each follow-up measurement separately, based on the question in the questionnaire assessing use of Move games (in hours per week) at T_1m_, T_4m_ and T_10m_. Again, two models are constructed for each outcome measure: *model 1* adjusts for baseline outcome measure; and *model 2* adjusts for demographics (age, sex, ethnicity and adolescent educational level) that are expected to associate with the outcome [3-5].

Additionally, a sensitivity analyses is performed by constructing all the models excluding the more extreme values on sedentary screen time (e.g. > 12 hours per day of non-active time). Further, if feasible (i.e. if the number of participants allows), explorative analyses are performed to check whether sex, age, adolescent educational level and ethnicity are effect modifiers, using a significance level of 0.10 because the study is not powered for detecting effect modification.

For the process evaluation, descriptive statistics are used describing the use of the Move games based on the Move game calendar over the ten months intervention period and the process evaluation measures. All statistical analyses are performed using IBM SPSS Statistics version 21 and statistical significance level is set at p < 0.05 for detecting main intervention effects and p < 0.10 for detecting effect modifiers.

## Discussion

This paper describes the design of a trial to test the effects of a family oriented active game intervention on anthropometrics and self-reported behavior among adolescents. As mentioned in the introduction, the current trial on active games contributes to existing literature because it is one of the few adequately powered trials, includes normal weight adolescents, has a relatively long follow up period (ten months) and includes a comprehensive process evaluation. Further, the intervention uses the newest generation of active games and incorporates several motivational elements to encourage (maintenance of) active game play. Several studies have focused on overweight/obese children and showed that an active gaming intervention can result in small effects on BMI or body weight [35,60]. However, as far as we know the current trial is the first well powered RCT including normal weight adolescents. Furthermore, the comprehensive process evaluation will provide important insight into whether adolescents adhere to the active game intervention over a ten month period, whether they enjoy playing active games and the factors that hinder or encourage them to play the active games. This information can help to make future active game interventions more effective.

For the current study we chose to encourage the adolescents in the intervention group to substitute non-active gaming with active gaming as much as possible but at least one hour per week. The potential contribution of active games on prevention of excessive weight gain in adolescents, lies partly in the fact that adolescents are intrinsically motivated to play video games and active game interventions capitalize on this intrinsic motivation. As stated before behavior change is more likely to be maintained if one is intrinsically motivated, which means that one enacts in an activity because of the activity itself, because one enjoys the activity and not because of external factors [25]. Giving adolescents an encouragement to play the active games a minimum amount of time might conflict with this potential intrinsic motivation for active game play. However, it is suggested that only providing active games is not enough to impact physical activity or body weight and it is important to also give an instruction regarding the use of the active games [46,48].

The encouragement for the intervention group to replace at least one hour per week of non-active gaming with active gaming, is based on calculations in adults demonstrating that excessive weight gain can be prevented if the energy balance is affected by 70 kcal per week. This was the best estimation that could be made when designing the study, because no child specific equation was available at that time. Recently, Hall et al. [61] developed and validated a model that quantifies the energy imbalance underlying excess bodyweight in children and calculates the necessary intervention magnitude to achieve bodyweight change in children. This model shows that around 116 extra kcal per week should be expended to prevent an excessive weight gain of 0.5 kg in children with an average age of 14 years old. As studies have shown that active gaming can elicit an energy expenditure of 381 kcal [23], one hour of active gaming (assuming it does not replace more vigorous activities such as sports) should in theory be sufficient to prevent excess weight gain in children.

A concern regarding promoting active game play is that active game play might replace traditional forms of physical activity (e.g. cycling, playing outside) because these activities elicit greater energy expenditure and may therefore contribute more to prevention of excessive weight gain than active gaming. In a descriptive Dutch study among active gaming adolescents and their parents, adolescents reported that they thought active gaming had replaced sedentary activities [45]. However, this was asked in retrospect after adolescents were already playing active games, and recall bias might have occurred. To get more insight in whether stimulating active gaming will lead to replacement of sedentary activities or whether it takes adolescents away from physical activities in which they already participate, the current study also evaluates the effect of providing active games on sedentary behavior and physical activity.

The present study is subject to some limitations that need to be acknowledged. Although we obtain the primary outcome measures objectively (e.g. adolescents’ height, weight, waist/hip circumference and skinfolds), the secondary outcome measures (e.g. adolescents’ physical activity, sedentary behavior, consumption of snacks and sugar-sweetened beverages) are self-reported and thus liable to social desirability and recall bias. However, this will occur in both groups resulting in potential bias towards the null.

In conclusion, this trial will provide important new information on the potential contribution of active games to the prevention of excessive weight gain in adolescents and the process evaluation will provide relevant information for future active games studies on factors hindering or promoting active game play.

## Competing interests

The authors declare that they have no financial relationships relevant to this article. Sony Benelux provides the PlayStation Move packages and video games for the study participants, but did not have any role in the design, conduct or analysis of the study. Funding for the study was obtained from The Netherlands Organization for Health Research and Development.

## Authors’ contributions

MS, EdV, MC, JB and JS designed the study. MS and MB conducted the sample size calculation and designed the statistical analyses plan. All authors read and approved the analyses plan. MvB wrote a first draft of the manuscript, MS drafted the manuscript further and EdV, MC, JB and JS critically revised the manuscript. All authors read and approved the final manuscript.

## Pre-publication history

The pre-publication history for this paper can be accessed here:

http://www.biomedcentral.com/1471-2458/14/275/prepub

## References

[B1] Livingstone MB Childhood obesity in Europe: a growing concern Public Health Nutr 2001 4 1A 109 116 11255500 10.1079/phn2000106

[B2] Ogden CL Carroll MD Curtin LR McDowell MA Tabak CJ Flegal KM Prevalence of overweight and obesity in the United States, 1999–2004 JAMA 2006 295 13 1549 1555 10.1001/jama.295.13.1549 16595758

[B3] Schönbeck Y Talma H van Dommelen P Bakker B Buitendijk SE Hirasing RA van Buuren S Increase in prevalence of overweight in Dutch children and adolescents: a comparison of nationwide growth studies in 1980, 1997 and 2009 PLoS One 2011 6 11 e27608 10.1371/journal.pone.0027608 22110687 PMC3216980

[B4] Brug J van Stralen MM te Velde SJ te Velde SJ Chinapaw MJ de Bourdeaudhuij I Lien N Bere E Maskini V Singh AS Maes L Moreno L Jan N Kovacs E Lobstein T Manios Y Differences in weight status and energy-balance related behaviors among schoolchildren across Europe: the ENERGY-project PLoS One 2012 7 4 e34742 10.1371/journal.pone.0034742 22558098 PMC3338827

[B5] Brug J van Stralen MM Chinapaw MJ de Bourdeaudhuij I Lien N Bere E Singh AS Maes L Moreno L Jan N Kovacs E Lobstein T Manios Y te Velde SJ Differences in weight status and energy-balance related behaviours according to ethnic background among adolescents in seven countries in Europe: the ENERGY-project Pediatr Obes 2012 7 5 399 411 10.1111/j.2047-6310.2012.00067.x 22730265

[B6] Bell LM Curran JA Byrne S Roby H Suriano K Jones TW Davis EA High incidence of obesity co-morbidities in young children: a cross-sectional study J Paediatr Child Health 2011 47 12 911 917 doi:10.1111/j.1440-1754.2011.02102.x. Epub 2011 Sep 9 10.1111/j.1440-1754.2011.02102.x 21902753

[B7] Wang YC McPherson K Marsh T Gortmaker SL Brown M Health and economic burden of the projected obesity trends in the USA and the UK Lancet 2011 378 9793 815 825 10.1016/S0140-6736(11)60814-3 21872750

[B8] Withrow D Alter DA The economic burden of obesity worldwide: a systematic review of the direct costs of obesity Obes Rev 2011 12 131 141 10.1111/j.1467-789X.2009.00712.x 20122135

[B9] Alberga AS Sigal RJ Goldfield G Prud’homme D Kenny GP Overweight and obese teenagers: why is adolescence a critical period? Pediatric Obesity 2012 7 4 261 273 10.1111/j.2047-6310.2011.00046.x 22461384

[B10] Kvaavik E Tell GS Klepp KI Predictors and tracking of body mass index from adolescence into adulthood: follow-up of 18 to 20 years in the Oslo Youth Study Arch Pediatr Adolesc Med 2003 157 12 1212 1218 10.1001/archpedi.157.12.1212 14662578

[B11] Hill JO Understanding and addressing the epidemic of obesity: an energy balance perspective Endocr Rev 2006 27 7 750 761 10.1210/er.2006-0032 17122359

[B12] Tremblay MS LeBlanc AG Kho ME Saunders TJ Larouche R Colley RC Goldfield G Connor Gorber S Systematic review of sedentary behaviour and health indicators in school-aged children and youth Int J Behav Nutr Phys Act 2011 8 98 10.1186/1479-5868-8-98 21936895 PMC3186735

[B13] Boone JE Gordon-Larsen P Adair LS Popkin BM Screen time and physical activity during adolescence: longitudinal effects on obesity in young adulthood Int J Behav Nutr Phys Act 2007 4 26 10.1186/1479-5868-4-26 17559668 PMC1906831

[B14] te Velde SJ de Bourdeaudhuij I Thorsdottir I Rasmussen M Hagströmer M Klepp KI Brug J Patterns in sedentary and exercise behaviors and associations with overweight in 9–14 year old boys and girls: a cross sectional study BMC Public Health 2007 7 16 10.1186/1471-2458-7-16 17266745 PMC1800840

[B15] Stettler N Signer TM Suter PM Electronic games and environmental factors associated with childhood obesity in Switzerland Obes Res 2004 12 6 896 903 10.1038/oby.2004.109 15229327

[B16] Utter J Neumark-Sztainer D Jeffery R Story M Couch potatoes or french fries: are sedentary behaviors associated with body mass index, physical activity, and dietary behaviors among adolescents? J Am Diet Assoc 2003 103 10 1298 1305 10.1016/S0002-8223(03)01079-4 14520247

[B17] Lambiase M Treating pediatric overweight through reductions in sedentary behavior: a review of the literature J Pediatr Health Care 2009 23 1 29 36 doi:10.1016/j.pedhc.2008.04.005. Epub 2008 Aug 9. Review 10.1016/j.pedhc.2008.04.005 19103404

[B18] Rideout VJ Foehr UG Roberts DF Generation M2: media in the lives of 8- to 18-year olds 2010 Menlo Park, CA: Henry J. Kaiser Family Foundation

[B19] Simons M de Vet E Brug J Seidell J Chinapaw M Active and non-active video gaming among Dutch adolescents: who plays and how much? J Sports Sci Med 2013 doi:10.1016/j.jsams.2013.10.250 10.1016/j.jsams.2013.10.250 24275124

[B20] Nikken P Computerspellen in het gezin [Computergames in the family] 2003 Hilversum: NICAM

[B21] The Netherlands National Gamers Survey 2009 Available at: http://www.tns-nipo.com/kenniscentrum/nieuws/nederland-telt-9,3-miljoen-gamers/. Accessed December 348 6, 2010

[B22] Simons M de Vet E Hoornstra S Brug J Seidell J Chinapaw M Adolescents’views on active and non-active videogames: a focus group study Games Health J 2012 1 3 211 218 10.1089/g4h.2011.0032 26193439

[B23] Barnett A Cerin E Baranowski T Active video games for youth: a systematic review J Phys Act Health 2011 8 5 724 737 21734319 10.1123/jpah.8.5.724

[B24] Biddiss E Irwin J Active video games to promote physical activity in children and youth: a systematic review Arch Pediatr Adolesc Med 2010 164 7 664 672 20603468 10.1001/archpediatrics.2010.104

[B25] Ryan RM Deci EL Self-determination theory and the facilitation of intrinsic motivation, social development, and well-being Am Psychol 2000 55 1 68 78 11392867 10.1037//0003-066x.55.1.68

[B26] Ryan RM Deci EL Self-regulation and the problem of human autonomy: does psychology need choice, self-determination, and will? J Pers 2006 74 6 1557 1585 10.1111/j.1467-6494.2006.00420.x 17083658

[B27] Chaput JP Visby T Nyby S Klingenberg L Gregersen NT Tremblay A Astrup A Sjodin A Video game playing increases food intake in adolescents: a randomized crossover study Am J Clin Nutr 2011 93 6 1196 1203 10.3945/ajcn.110.008680 21490141

[B28] Oldham-Cooper RE Hardman CA Nicoll CE Rogers PJ Brunstrom JM Playing a computer game during lunch affects fullness, memory for lunch, and later snack intake Am J Clin Nutr 2011 93 2 308 313 10.3945/ajcn.110.004580 21147857

[B29] Kremers SP van der Horst K Brug J Adolescent screen-viewing behaviour is associated with consumption of sugar-sweetened beverages: the role of habit strength and perceived parental norms Appetite 2007 48 3 345 350 10.1016/j.appet.2006.10.002 17126451

[B30] Epstein LH Roemmich JN Paluch RA Raynor HA Influence of changes in sedentary behavior on energy and macronutrient intake in youth Am J Clin Nutr 2005 81 2 361 366 15699222 10.1093/ajcn.81.2.361

[B31] Epstein LH Saad FG Giacomelli AM Roemmich JN Effects of allocation of attention on habituation to olfactory and visual food stimuli in children Physiol Behav 2005 84 2 313 319 10.1016/j.physbeh.2004.12.009 15708783

[B32] Lyons EJ Tate DF Ward DS Wang X Energy intake and expenditure during sedentary screen time and motion-controlled video gaming Am J Clin Nutr 2012 96 2 234 239 10.3945/ajcn.111.028423 22760571 PMC3396440

[B33] Hopkins M King NA Blundell JE Acute and long term effects of exercise on appetite control: is there any benefit for weight control? Curr Opin Clin Nutr Metab Care 2010 13 635 640 10.1097/MCO.0b013e32833e343b 20717015

[B34] Mellecker RR Lanningham-Foster L Levine JA McManus AM Energy intake during activity enhanced video game play Appetite 2010 55 2 343 347 10.1016/j.appet.2010.07.008 20655345

[B35] Maddison R Foley L Ni Mhurchu C Jiang Y Jull A Prapavessis H Hohepa M Rodgers A Effects of active video games on body composition: a randomized controlled trial Am J Clin Nutr 2011 94 1 156 163 10.3945/ajcn.110.009142 21562081

[B36] Straker LM Abbott RA Smith AJ To remove or to replace traditional electronic games? A crossover randomised controlled trial on the impact of removing or replacing home access to electronic games on physical activity and sedentary behaviour in children aged 10–12 years BMJ Open 2013 3 6 doi:10.1136/bmjopen-2013-002629. Print 2013 10.1136/bmjopen-2013-002629 PMC3686216 23818650

[B37] Maloney AE Bethea TC Kelsey KS Marks JT Paez S Rosenberg AM Catellier DJ Hamer RM Sikich L A pilot of a video game (DDR) to promote physical activity and decrease sedentary screen time Obesity (Silver Spring) 2008 16 9 2074 2080 doi:10.1038/oby.2008.295 10.1038/oby.2008.295 19186332

[B38] Ni Mhurchu C Maddison R Jiang Y Jull A Prapavessis H Rodgers A Couch potatoes to jumping beans: a pilot study of the effect of active video games on physical activity in children Games for Health Journal 2012 1 2 96 103 doi:10.1089/g4h.2011.0009 10.1089/g4h.2011.0009 PMC2254648 18257911

[B39] Maloney AE Threlkeld KA Cook WL Comparative effectiveness of a 12-week physical activity intervention for overweight and obese youth: exergaming with dance dance revolution Games Health J 2012 1 2 96 103 doi:10.1089/g4h.2011.0009 10.1089/g4h.2011.0009 26193183

[B40] Madsen KA Yen S Wlasiuk L Newman TB Lustig R Feasibility of a dance videogame to promote weight loss among overweight children and adolescents Arch Pediatr Adolesc Med 2007 161 1 105 107 17199076 10.1001/archpedi.161.1.105-c

[B41] Chinapaw MJM Jacobs WM Vaessen EPG Titze S van Mechelen W The motivation of children to play an active video game J Sci Med Sport 2008 11 2 163 166 10.1016/j.jsams.2007.06.001 17706461

[B42] LeBlanc AG Chaput JP McFarlane A Colley RC Thivel D Biddle SJ Maddison R Leatherdale ST Tremblay MS Active video games and health indicators in children and youth: a systematic review PLoS One 2013 8 6 e65351 doi:10.1371/journal.pone.0065351. Print 2013 10.1371/journal.pone.0065351 23799008 PMC3683002

[B43] NHS-NRG Preventie van gewichtsstijging en richtlijnen voor gewichtsbeheersing in Nederland 2007 http://www.fontysmediatheek.nl/w/images/9/95/NHS-NRGRichtlijnenGewichtsbeheersing.pdf

[B44] Paez S Maloney A Kelsey K Wiesen C Rosenberg A Parental and environmental factors associated with physical activity among children participating in an active video game Pediatr Phys Ther 2009 21 3 245 253 10.1097/PEP.0b013e3181b13a82 19680066

[B45] Simons M Bernaards C Slinger J Active gaming in Dutch adolescents: a descriptive study Int J Behav Nutr Phys Act 2012 9 118 doi:10.1186/1479-5868-9-118 10.1186/1479-5868-9-118 23031076 PMC3493303

[B46] Baranowski T Abdelsamad D Baranowski J O’Connor TM Thompson D Barnett A Cerin E Chen TA Impact of an active video game on healthy children’s physical activity Pediatrics 2011 129 3 e636 e642 22371457 10.1542/peds.2011-2050 PMC3289528

[B47] Daley AJ Can exergaming contribute to improving physical activity levels and health outcomes in children? Pediatrics 2009 124 763 771 originally published online Jul 13, 2009 10.1542/peds.2008-2357 19596728

[B48] Peng W Crouse JC Lin J-H Using active video games for physical activity promotion: a systematic review of the current state of research Health Educ Behav 2013 40 171 originally published online 6 July 2012 10.1177/1090198112444956 22773597

[B49] Durnin JV Rahaman MM The assessment of the amount of fat in the human body from measurements of skinfold thickness Br J Nutr 1967 21 3 681 689 10.1079/BJN19670070 6052883

[B50] de Jong E Visscher TL Hirasing RA Heymans MW Seidell JC Renders CM Association between TV viewing, computer use and overweight, determinants and competing activities of screen time in 4- to 13- year-old children Int J Obes 2013 37 47 53 doi:10.1038/ijo.2011.244; published online 13 December 2011 10.1038/ijo.2011.244 22158265

[B51] Philippaerts RM Matton L Wijndaele K Balduck AL de Bourdeaudhuij I Lefevre J Validity of a physical activity computer questionnaire in 12- to 18-year-old boys and girls Int J Sports Med 2006 27 2 131 136 10.1055/s-2005-837619 16475059

[B52] van der Horst K Oenema A van de Looij-Jansen P Brug J The ENDORSE study: research into environmental determinants of obesity related behaviors in Rotterdam schoolchildren BMC Public Health 2008 8 142 doi:10.1186/1471-2458-8-142 10.1186/1471-2458-8-142 18442370 PMC2413223

[B53] van Assema P Brug J Ronda G Steenhuis I The relative validity of a short Dutch questionnaire as a means to categorize adults and adolescents to total and saturated fat intake J Hum Nutr Diet 2001 14 5 377 390 10.1046/j.1365-277X.2001.00310.x 11906579

[B54] McAuley E Duncan T Tammen VV Psychometric properties of the intrinsic motivation inventory in a competitive sport setting: a confirmatory factor analysis Res Q Exerc Sport 1987 60 1 48 58 2489825 10.1080/02701367.1989.10607413

[B55] Davis FD Perceived usefulness, perceived ease of Use, and user acceptance of information technology MIS Quarterly 1989 13 3 319 340 10.2307/249008

[B56] Hsu C-L Lu H-P Why do people play on-line games? An extended TAM with social influences and flow experience Inf Manage 2004 41 7 853 868 10.1016/j.im.2003.08.014

[B57] Francis JJ Eccles MP Johnston M Walker A Grimshaw J Foy R Kaner EFS Smith L Bonetti D Constructing questionnaires based on the theory of planned behavior: a manual for health services researchers 2004 United Kingdom: Centre for Health Services Research: Newcastle Upon Tyne

[B58] Singh AS Chin A Paw MJ Kremers SP Visscher TL Brug J van Mechelen W Design of the Dutch obesity intervention in teenagers (nrg-doit): systematic development, implementation and evaluation of a school based intervention aimed at the prevention of excessive weight gain in adolescents BMC Public Health 2006 6 304 10.1186/1471-2458-6-304 17173701 PMC1769372

[B59] Ainsworth BE Haskell WL Whitt MC Irwin ML Swartz AM Strath SJ O’Brien WL Bassett DR Jr Schmitz KH Emplaincourt PO Jacobs DR Jr Leon AS Compendium of physical activities: an update of activity codes and MET intensities Med Sci Sports Exerc 2000 32 9 Suppl S498 S504 10993420 10.1097/00005768-200009001-00009

[B60] Staiano AE Abraham AA Calvert SL Adolescent exergame play for weight loss and psychosocial improvement: a controlled physical activity intervention Obesity (Silver Spring) 2013 21 3 598 601 doi:10.1002/oby.20282 10.1002/oby.20282 23592669 PMC3473097

[B61] Hall KD Butte NF Swinburn BA Chow CC Dynamics of childhood growth and obesity: development and validation of a quantitative mathematical model Lancet Diab Endocrinol 2013 1 97 105 10.1016/S2213-8587(13)70051-2 PMC3857695 24349967

